# Genetic, metabolite and developmental determinism of fruit friction discolouration in pear

**DOI:** 10.1186/s12870-014-0241-3

**Published:** 2014-09-16

**Authors:** Munazza Saeed, Lester Brewer, Jason Johnston, Tony K McGhie, Susan E Gardiner, Julian A Heyes, David Chagné

**Affiliations:** Centre for Postharvest & Refrigeration Research, Massey University, Private Bag 11 222, Palmerston North, 4442 New Zealand; The New Zealand Institute for Plant & Food Research Limited (Plant & Food Research), Private Bag 11600, Palmerston North, 4442 New Zealand; Plant & Food Research, Motueka Research Centre, Old Mill Road, Motueka, 7198 New Zealand; Plant & Food Research, Hawkes Bay Research Centre, Private Bag 1401, Havelock North, New Zealand

**Keywords:** Friction discolouration, Postharvest, QTL, SNP, PPO, *Pyrus*

## Abstract

**Background:**

The unattractive appearance of the surface of pear fruit caused by the postharvest disorder friction discolouration (FD) is responsible for significant consumer dissatisfaction in markets, leading to lower returns to growers. Developing an understanding of the genetic control of FD is essential to enable the full application of genomics-informed breeding for the development of new pear cultivars. Biochemical constituents [phenolic compounds and ascorbic acid (AsA)], polyphenol oxidase (PPO) activity, as well as skin anatomy, have been proposed to play important roles in FD susceptibility in studies on a limited number of cultivars. However, to date there has been no investigation on the biochemical and genetic control of FD, employing segregating populations. In this study, we used 250 seedlings from two segregating populations (POP369 and POP356) derived from interspecific crosses between Asian (*Pyrus pyrifolia* Nakai and *P. bretschneideri* Rehd.) and European (*P. communis*) pears to identify genetic factors associated with susceptibility to FD.

**Results:**

Single nucleotide polymorphism (SNP)-based linkage maps suitable for QTL analysis were developed for the parents of both populations. The maps for population POP369 comprised 174 and 265 SNP markers for the male and female parent, respectively, while POP356 maps comprised 353 and 398 SNP markers for the male and female parent, respectively. Phenotypic data for 22 variables were measured over two successive years (2011 and 2012) for POP369 and one year (2011) only for POP356. A total of 221 QTLs were identified that were linked to 22 phenotyped variables, including QTLs associated with FD for both populations that were stable over the successive years. In addition, clear evidence of the influence of developmental factors (fruit maturity) on FD and other variables was also recorded.

**Conclusions:**

The QTLs associated with fruit firmness, PPO activity, AsA concentration and concentration of polyphenol compounds as well as FD are the first reported for pear. We conclude that the postharvest disorder FD is controlled by multiple small effect QTLs and that it will be very challenging to apply marker-assisted selection based on these QTLs. However, genomic selection could be employed to select elite genotypes with lower or no susceptibility to FD early in the breeding cycle.

**Electronic supplementary material:**

The online version of this article (doi:10.1186/s12870-014-0241-3) contains supplementary material, which is available to authorized users.

## Background

Consumer awareness of the long-term health benefit of fruit consumption has significantly increased the demand for high-quality fresh fruit. Postharvest disorders of fresh fruits are a major factor contributing to product deterioration and crop losses. Many such disorders are physiological in origin and may be related to time of harvest (maturity), season and growing region, as well as cultivar (genotype). A range of postharvest treatments is traditionally employed to minimise disorders and typically a single treatment is not enough to control a postharvest disorder. However, new technologies in molecular genetics and metabolomics now enable us to dissect the control of fruit postharvest disorders into fine-scale determinants that include biochemical and genetic controls and provide some hope for development of more targeted solutions.

Friction discolouration (FD) is a serious postharvest disorder in pear generally categorised by diffuse brown skin marks that occur as a result of mechanical damage and lead to the enzymatic browning of the fruit skin. Mechanical damage can occur at any step during picking, sorting, processing or transportation [1,2]. FD is different from bruising as it is confined only to epidermal layers, with no damage to flesh. Although nutritive value and flavour is not affected by FD, the unattractive appearance of the fruit results in consumer dissatisfaction and reduced prices, which can lead to severe market losses [3,4]. Mechanical injury also enhances respiration and moisture loss from the injured area, as well as ethylene production, a combination that may speed ripening and reduce shelf life.

The underlying mechanism behind FD involves a combination of physical stress and biochemical reactions, in particular the enzymatic oxidation of polyphenols by polyphenol oxidase (PPO) [5]. Polyphenols are the specific substrates for the underlying browning reaction in FD, with cholorogenic acid being the most abundant phenolic compound in pears and generally believed to play the most crucial role in enzymatic browning [5]. Other factors may also contribute to differential FD susceptibility, such as the activity of PPO and the concentration of ascorbic acid (AsA) as a key inhibitor to enzymatic browning [6,7]. Additionally, the nature of the skin surface may influence its susceptibility to physical damage [8]. Some varieties of pear are known to be more or less susceptible to FD, indicating underlying genetic determination, but still, fruit susceptibility can vary with the maturation stage of a single cultivar. It also has been noted that European species are less susceptible than Asian species in general [9-13]. The Plant & Food Research (PFR) pear breeding programme ([14]) applies strong selection pressure against FD [15] and this process would be greatly benefitted by the application of marker-assisted selection (MAS) at the seedling stage, to enable selection of genotypes with low genetic potential to develop FD. However, the detailed phenotypic analysis required for trait association with genetic markers has seldom been undertaken in pear for any fruit quality parameters, let alone one as complex as FD.

Quantitative trait loci (QTLs) for fruit traits, such as fruit shape, sugar content, acid content, vitamin C content, maturity, and fruit skin composition have been mapped in a range of fruit crops, including tomato [1,16-18], peach [19-21], apple [22-27], strawberry [28], sweet cherry [29,30], apricot [31,32] and papaya [33], among others. There is a single report on QTL analysis of pear fruit characters by Zhang et al. [34], in which QTLs for traits such as fruit weight, diameter, length, soluble solid content, fruit shape index, and maturity date were identified in Chinese pear (*P. bretschneideri*) cultivars ‘Bayuehong’ and ‘Dangshansuli’. There are two reports in apple [35,36] and one in melon [37] evaluating the QTLs associated to fruit physiological disorders, however, none of them has used systematic approach to evaluate the genomic regions (QTLs) linked to disorder as well as characters influencing fruit. Also, our study is the first focusing on genetic solution to a postharvest disorder in pear.

A number of genetic maps for pear have been developed for the purpose of trait mapping, using a range of molecular markers, including RAPDs (random amplified polymorphic DNA) [38], AFLPs (amplified fragment length polymorphism), SSRs (simple sequence repeats) [39-43] and sequence-related amplified polymorphisms (SRAPs) [44]. However, none of these maps has been developed directly from *Pyrus* genome sequences. Recently, Wu et al. [45] used next generation sequencing to develop a dense interspecific genetic map of ‘Bayuehong’ (*P. bretschneideri* × *P. communis*) × ‘Dangshansuli’ (*P. bretschneideri*) comprising 2005 SNP (single nucleotide polymorphism) markers, to anchor the Chinese pear genome. However, there are no reports to date of trait mapping in this population using these 2005 SNPs. More recently, the International RosBREED SNP Consortium (IRSC) Illumina Infinium® II 9K apple and pear SNP chip [46,47] was developed for genetic mapping of traits in five segregating populations of pear, including two interspecific populations segregating for FD.

Although there are previous studies on postharvest aspects of FD [4,48-51], there has been no attempt to explore systematically the genetic basis and control of this disorder. Hence we have focussed first on developing an in-depth understanding of the variation of phenotypes that might be associated with FD development (FD intensity, firmness, total soluble solids, PPO activity, and concentration AsA and seventeen polyphenols) among the different genotypes in our mapping populations. Here the goal is to identify factors that might influence the development of FD and hence differential susceptibility to this disorder. Breeders have reported a high narrow sense heritability (0.72) among inter-specific pear breeding populations, including both populations under study, which suggested that comprehensive genetic gain could be obtained for FD [15]. Our strategy utilises this phenotypic analysis for subsequent QTL analysis to identify genetic loci associated with FD, utilising two related populations in which individuals segregated for susceptibility to FD. Our study is the first report of the use of a SNP-based dense genetic linkage map for QTL analysis in pear, as well as the first systematic investigation of the genetic control of a postharvest disorder in pear.

## Methods

### Plant material and fruit sampling

**Figure 1:**
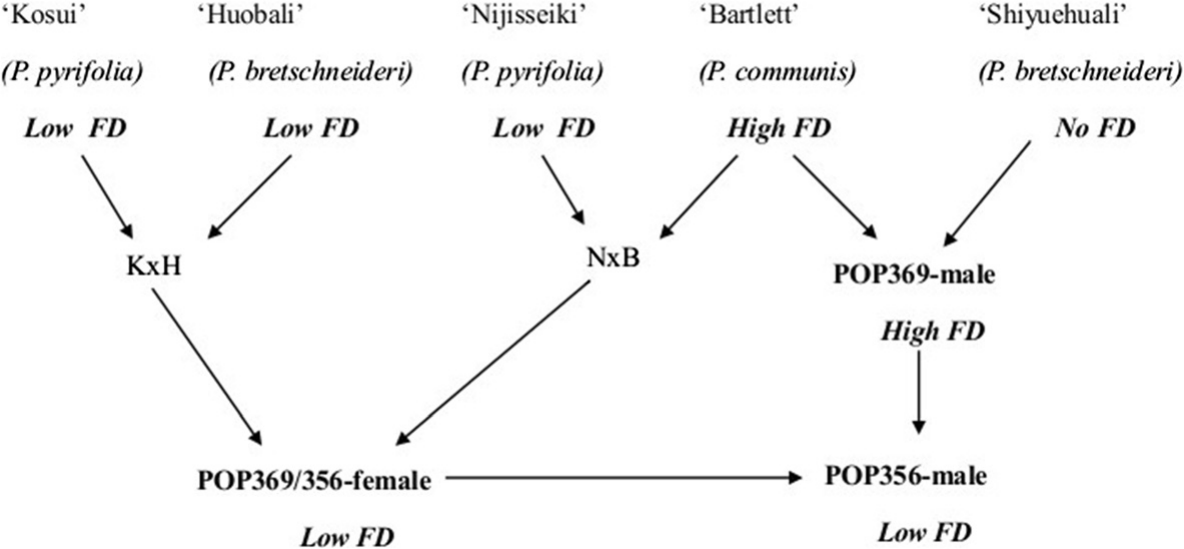
**Genetic information and friction discolouration (FD) potential concerning the parents of POP369 and POP356 pear populations.**

Two full-sib families (POP356 and POP369) resulting from interspecific crosses between Asian (*P. pyrifolia* Nakai and *P. bretschneideri* Rehd.) and European pears (*P. communis* L.) (Figure 1) were grown at the Motueka Research Centre, PFR, Motueka, New Zealand. POP369 is a population of 1028 full-sib genotypes from a cross between POP369-female and POP369-male. Both families were planted on their own roots into the orchard in 2007 at row spacing of 3 m and in row spacing of 0.75 m. Plants received a standard fertiliser programme and any branches at least one meter above the wire trellis at a height of 1.8m were bent down to the wire in January each year. Trees that had not commenced fruiting were girdled in December with a Vaca cane girdler, which removed a 4 mm horizontal strip of vascular tissue below the 1.8 m wire. Fruit from 98 seedlings from the POP369 population were harvested for phenotype and QTL analysis in 2011 and 2012. POP356 was a population totaling 1285 full-sib genotypes from a cross between POP356-female and POP356-male parent. Fruit from 143 seedlings from the POP356 population were collected for phenotype and QTL analysis in 2011. Fruit harvest for each genotype began when fruit had a green-yellow background colour and were harvested every 7–12 days until fruit ran out. Fruit was stored for 90 to 100 days at 3°C for initiation of ripening, and then transported to PFR Palmerston North by refrigerated truck for further analysis.

### Friction discolouration assessment

**Figure 2:**
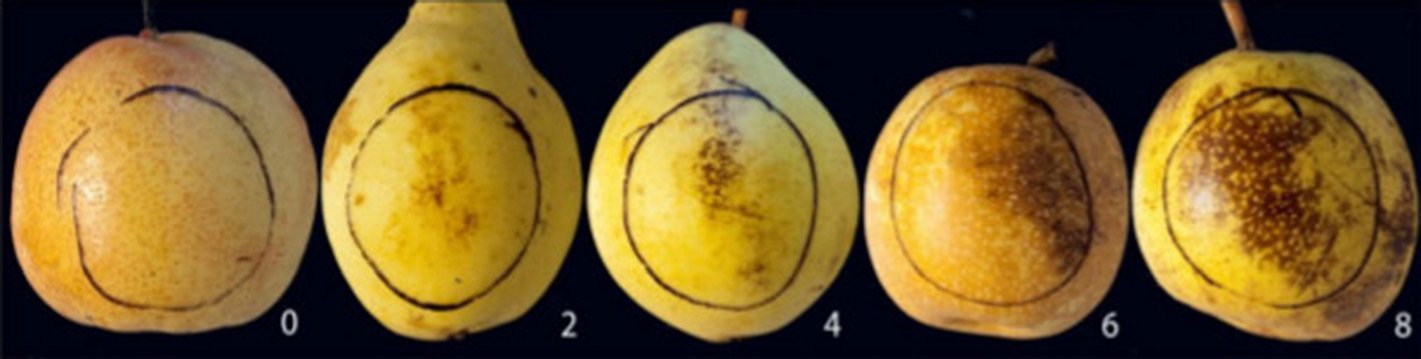
**Visual scale for friction discolouration (FD) score assessment in pear.**

To assess FD, four random fruit were selected per seedling, removed from the cool store and kept overnight at room temperature. FD was induced the next day by rubbing the fruit twice against a fiber tray cup surface [15,52]. After another 24 h at room temperature, browning area and intensity was recorded on a 0–9 scale, where 0 is absence of FD and 9 is the highest FD score (Figure 2). FD was scored by the same single assessor in 2011 and 2012 to reduce experimental error. FD score was averaged across all four fruit for each seedling and harvest date.

### Total soluble solids and firmness

Total soluble solids content (TSS) and fruit firmness were measured using two of the same fruit on which FD was determined. Equal amounts of juice from both ends of the fruit were used to assess TSS (°Brix) using a digital refractometer (Atago, Japan). Compression firmness was measured in Newton (N) at two points separated by 180° around each fruit equator, using a texture analyser TA-XT Plus (Stable Micro System, Godalming, UK) fitted with a 7.9 mm probe. TSS and fruit firmness was averaged across both fruits and harvest date for each seedling.

### Peel sample preparation and extraction for polyphenol and AsA quantification

Peel of 1 mm thickness, consisting of fruit skin with underlying flesh was removed from the equatorial area of 4–5 fruit per genotype (preferably same fruits that were used for FD assessment), snap frozen and then ground together with a pellet of dry ice, using a coffee grinder. Ground peel tissue was stored frozen at −80°C until further analysis. Extraction solution (80:20:1 EtOH: H_2_O: formic acid; 5 ml) was added to 1 g of finely ground peel and left for 24 h at 4°C. After 24 h, culture tubes containing the extract were centrifuged (1000 g, 15 min) at 20 ± 2°C and the extracts were sampled directly into high performance liquid chromatography (HPLC) vials. Aliquots of the extracts were diluted in cold solvent (50:50:1 MeOH: H_2_O: formic acid) prior to polyphenol analysis.

### Polyphenol quantification in pear peel

Polyphenol content of these extracts was analysed using a liquid chromatography mass spectrometry (LC-MS) system that comprised a Dionex Ultimate® 3000 Rapid Separation LC system and a micrOTOF-QII mass spectrometer (Bruker Daltonics, Bremen, Germany) fitted with an electrospray source operated in negative mode. The analytical column was Zorbax™ SB-C18 HD, 2.1 × 150 mm, 1.8 μm (Agilent, Melbourne, Australia). Solvents used in 2011 were A = 90% methanol, and B = 0.5% formic acid in water (v/v), with a gradient of 5% A, 95% B for 0–0.5 min; gradient to 40% A, 60% B from 0.5-8 min; gradient to 75% A, 25% B from 8–11 min; and gradient to 100% A, 0.0% B from 11–12 min. The composition was then held at 100% A from 12–14 min; decreased down to 5.0% A, 95% B between 14–14.2 min. The gradient of 5% A, 95% B was maintained until injection of the next sample. Total run time for each sample was 17 minutes.

Solvents used in 2012 were A = 100% acetonitrile and B = 0.1% formic acid with a gradient of 5% A, 95% B for 0–0.5 min; gradient to 30% A, 70% B from 0.5-10 min; gradient to 100% A, 0.0% B from 10–14.50 min. The composition was then held at 100% A from 14.5-16.50 min; decreased to 5.0% A, 95% B between16.5-17 min, and maintained until the next sample was injected. Total run time for each sample was 20 minutes.

Polyphenolic components were quantified using QuantAnalysis (Bruker Daltonics, Bremen, Germany) by extracting accurate (±10 mDa) mass ion chromatograms. As external standards were not available for all the detected compounds, we used peak area (response/min) for calculations involving phenolic concentrations for all the compounds.

### Polyphenol oxidase activity quantification in pear peel

PPO activity was measured spectrophotometrically as described in [50,53] with a few modifications. Extraction solution (0.05 M phosphate buffer, 1MKCl, pH 7) and 1 g polyvinylpolypyrrolidone (PVPP) was added to 1 g finely ground frozen peel. This mixture was homogenised and centrifuged (14 000 g) for 15 minutes at 4°C. Each sample contained 25 μl extract, 220 μl reaction buffer (0.2 M phosphate, 0.1 M citrate, pH 6.5) and 55 μl standard catechol solution (0.5 M catechol in a 10-fold dilution of the reaction buffer). The assay procedure was carried out at 20°C with initial shaking for 2 sec. The increase in absorbance at 420 nm was then recorded by spectrophotometer (Molecular Devices Spectra Max Plus, Sunnyvale, CA, USA) with readings at 2 sec intervals, and eight samples read simultaneously. Enzyme activity was calculated from the initial 20 sec gradient of curves in 2011, and initial 30 sec in 2012. PPO activity is presented as the change in absorbance at 420 nm per gram fresh pear peel per minute (change in A_420_/ g/minute).

### Ascorbic acid quantification in pear peel

AsA content in pear fruit peel was quantified on an Alliance 2690 HPLC (Waters, Milford, MA, USA). Solvent-based peel extracts (prepared for polyphenol quantification) were diluted 1:4 with tris-(2-carboxyethyl) phosphine and incubated in the dark for 90 minutes. AsA was resolved using a Synergi 4 μm Hydro 4.6 × 250 mm (Phenomenex, Torrance, CA) reversed phase column protected with a guard column of the same packing. Column temperature was set at 40°C. The solvents used were A = 0.5% v/v phosphoric acid (98%) and C = 70:30 methanol/Milli-Q water (2%) with proportions remaining the same throughout the run. Sample injection volume was 10 μL and flow rate was 0.8 mL per minute. Total run time for each sample was set at 9 min isocratic run time. An external calibration curve was constructed for AsA based on three standards with concentrations 10 μg/mL, 20 μg/mL and 50 μg/mL, respectively. Quantification of AsA was based on peak areas determined at 240 nm in 2011, and 250 nm in 2012. Chromatographic data were collected and manipulated using a Chromeleon® Chromatography Management System version 6.8. The AsA concentration derived from the HPLC analysis was transformed from μg/mL (Cv) to μg/g (Cw) of fresh weight by dividing Cv by the fresh weight of the sample.

### Statistical analysis

Minitab® version 16.1.1 was used to test the trait distribution, to calculate the Pearson correlation of the traits, and to perform analysis of variance (ANOVA).

### DNA extraction and SNP screening

For the POP369 population, DNA from 94 full-sibs and the pollen parent was purified from young leaves using a CTAB (hexadecyltrimethylammonium bromide) extraction method [54], followed by column purification using the NucleoSpin® kit (Macherey-Nagel GmbH & Co. KG). For the POP356 population, DNA from 123 full-sibs and the pollen parent was extracted using the QIAGEN DNeasy Plant Kit (QIAGEN GmbH, Hilden, Germany). DNA could not be prepared from the female parent of POP369 and POP356 common to both populations as trees of this genotype no longer existed in the field. DNA quantifications were carried out using a NanoDrop™ 2000c spectrophotometer (Thermo Fisher Scientific Inc.).

Genomic DNA (200 ng) from progeny and male parents was amplified and hybridised to the apple and pear IRSC 9K SNP array [46,47] following the Illumina Infinium® HD Assay Ultra protocol (Illumina Inc., San Diego, CA, USA) and scanned with the Illumina iScan. Data was analysed using Illumina’s GenomeStudio v1.0 software Genotyping Module, setting a *GenCall* Threshold of 0.15. The software automatically determines the cluster positions of the AA/AB/BB genotypes for each SNP and displays them in normalised graphs. A systematic method was used for evaluating the SNP array data using quality metrics extracted from GenomeStudio (Illumina): GenTrain score ≥ 0.50, minor allelic frequency (MAF) ≥ 0.15 and call rate > 80%. The genotype of the female parent was inferred manually on the basis of the genotype of the other parent and progeny. SNPs that were highly distorted or which had the genotype of one or both parents missing were manually edited in GenomeStudio. Furthermore, the SNPs for which 25% and 50% of the individuals were not called in clusters were manually edited, since this could be due to null allele segregation.

### Genetic map construction and QTL mapping

The genetic maps of the four parents of the two populations were constructed using double pseudo test cross methodology [55] and JoinMap v3.0 software [56] based on the SNP data for the individuals in each population. Linkage groups were determined with a LOD score of 5 or higher for grouping and the Kosambi mapping function was used for genetic distance calculation.

Linkage group numbering was determined using apple SNPs [46] anchored to the reference genome of ‘Golden Delicious’. Furthermore POP369 shares a common parent with a population published earlier in [47] that has 54 simple sequence repeats mapped to enable LG numbering that is consistent with previously published pear and apple maps. The alignment of male parental maps from both populations is provided in Additional file 1: Figure S1.

The four parental maps were drawn and aligned using MapChart v2.2 [57] and QTL analysis was performed using MapQTL 5.0 [58]. For individual seedlings with more than one fruit harvest, both average and maximum score of the data were used as phenotypic data, where FD score was expressed for each individual as maximum FD and average FD. The data distribution for each compound was verified before the QTL analysis. QTLs were identified using the Kruskal-Wallis Test (KW) because most of the traits were not normally distributed. SNPs are presented using the NCBI dbSNP accession number (ss #) and SNPs with null alleles are represented with the prefix ‘null’.

## Results

### Friction discoloration variation in the pear segregating populations

Fruit from 241 individual genotypes were harvested in 2011 with some genotypes sampled multiple times – 206 fruit samples from 143 genotypes of family POP356 and 125 fruit samples from 98 genotypes of family POP369. In 2012, 177 fruit samples from 98 genotypes of the POP369 population were harvested, with multiple harvests where possible. In both years, fruit were assessed for FD, firmness, TSS, PPO activity, AsA and polyphenolic compounds concentrations. Means, medians, maxima and minima information for phenotypic traits averaged across multiple fruits and harvest date for each seedling are provided in Table 1. Both populations displayed a range of FD scores, from no FD observed for some genotypes, to high FD scores observed in other genotypes (Figure 2).

**Table 1:** **Ranges in trait data collected form pear populations POP369 and POP356**

	**POP369** **(2011)**				**POP369** **(2012)**				**POP356** **(2011)**			
**Trait**	**Min**	**Max**	**Median**	**Mean**	**Min**	**Max**	**Median**	**Mean**	**Min**	**Max**	**Median**	**Mean**
**Friction discolouration**	0	9	4	3.7	0.1	9	4.7	4.6	0	9	2	2.6
**Total soluble solids**	9.8	15.6	11.8	11.9	9	15.3	11.9	11.9	9.2	15.2	11.7	11.7
**Firmness**	10.3	54.2	31.5	31.7	13.9	48.6	23	23.9	14.2	55.8	29.5	29.8
**Polyphenol oxidase**	191.1	1424.7	761.1	779.1	24.2	258.1	77.2	93.4	103.3	1989	706.7	761.6
**Ascorbic Acid**	3.2	167.9	60.6	59.6	23.8	217.1	74.4	76.8	0	142.4	26.1	35.1
**Chlorogenic acid**	5341.8	112476.8	19664.6	24912.0	29503.7	175530.7	71581.6	78099.2	0	208083.1	42485.9	53134.6
**Cryptochlorogenic acid**	0	6330.3	1315.8	1539.8	1757	21934.2	6592.4	7739.5	104.4	10501.5	2390.3	2992
**Neochlorogenic acid**	0	3358.8	315.9	422.6	0	14282.5	1640.2	2000.4	0	4789.7	810.6	957.6
**Catechin**	0	2571	256.7	426.3	0	13644.8	1686.4	2514.4	0	4712.2	401.7	721.3
**Epicatechin**	1078	39207.9	7224.7	10392.7	11661.4	132773.5	38185.6	42373.9	0	111592.7	7709.7	14260.4
**Procyanidin dimer B2**	0	18355.3	4766.4	5366.5	4133.3	55342.1	20882	22486.3	0	46723.1	5424.3	8059.3
**Isorhamnetin 3**-**galactoside**	493.3	63829.6	10176.8	13770.6	1410.6	49696.3	8429.4	10913.7	0	90793.3	10818.2	17741.6
**Isorhamnetin 3**-**glucoside**					6114.4	121846.6	26221.3	31505.4				
**Isorhamnetin rutinoside**	788.7	18269.3	5258.3	6260.5	2649.5	36273.8	13589.1	14579.6	0	109860.2	11466.2	17960.3
***p***-**coumaryl quinic acid**	0	3507.2	0	297.1	0	10184.7	1067.1	1715.6	0	73981.9	1045.3	3709
**Quercetin arabinoside**	0	63039.5	7531.1	10031.2	58.5	75916.5	16686.7	19980.9	0	47628.8	6607.7	9347.8
**Quercetin galactoside**	0	14337.2	1033.1	2098	889.9	32231	6415.8	8207.6	0	41149.9	1449.9	3059.2
**Quercetin glucoside**	0	24575.7	5073.7	6732.8	4485.9	76754.7	19285	24123.3	0	79820.1	5774.5	8791
**Quercetin rutinoside**	0	4748.4	1042.4	1226.5	263.6	13591.8	3295.2	4033.3	0	27078.8	2159.5	3698.7
**Quercetin rhamnoside**	0	48041.9	222	1331	0	69621.9	234.1	14208.5	0	6158.7	189.6	475.9
**Quercetin**	0	3380.2	944	1046.6	0	74.7	0	12.7	0	3484.5	639.8	732.5
**comp**_**417.12** **(1)**	0	72548.3	6036.8	9254.7	0	62882.9	11794.7	14270.2	0	60209.4	7794.5	10971.6
**comp**_**417.12** **(2)**	0	32815.3	2874.7	4656.9	0	95921.3	18958.1	24810	0	25253.4	3469.8	4843.4

Data from POP369 were collected in two successive years (2011 and 2012) while POP356 was analysed in 2011 only. Ranges are collected from genotypes scores averaged across the harvests. Units for trait studied are following: FD (scale), TSS (°Brix), Firmness (N: newton), PPO (change in A_420_/g/minute), AsA and polyphenols compounds (concentration). N.B.: comp_417.12 (1) and comp_417.12 (2) are unknown polyphenols compounds identified from LC-MS quantification analysis, represented by their molecular weight.

**Figure 3:**
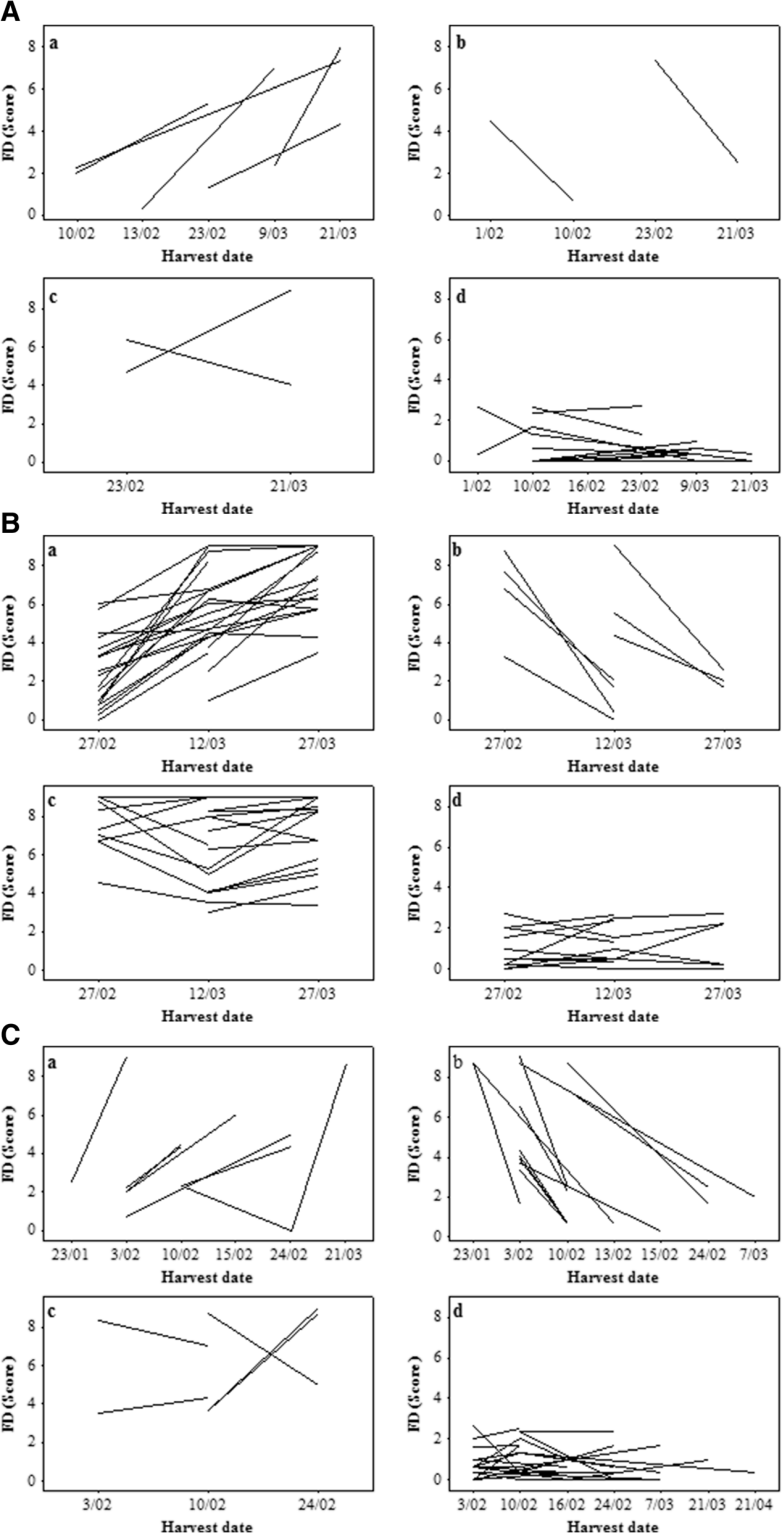
**Mean friction discolouration (FD) scores arranged by harvest dates for multiple harvests of genotypes. A)** Mean FD scores arranged by harvest date for POP369 in 2011, **B)** Mean FD scores arranged by harvest date for POP369 in 2012, **C)** Mean FD scores arranged by harvest date for POP356 in 2011. Genotypes with multiple harvests for individual tree were divided into four distinct groups a) represents the seedlings with increasing FD trend during the season b) represents seedlings with decreasing trends c) represents seedlings with consistent high FD susceptibility d) represents consistent low FD susceptibility.

Having more than one harvest from some genotypes provided the opportunity for comparison of the FD susceptibility between fruit at different stages of maturity. FD susceptibility showed substantial variation between different harvests, genotypes and even between fruit of the same genotype (Figure 3A,B,C). Both populations showed a variety of trends for FD incidence with different harvest dates for same genotypes. Of 23 genotypes with multiple harvests in 2011, five genotypes of the POP369 population exhibited low FD at early harvests and high FD at later harvests. However, within the same segregating population, two genotypes exhibited a decrease in FD susceptibility later in the season. There were 16 genotypes that did not show any variation of FD score between the harvest dates, with 14 genotypes scoring consistently low, and two exhibiting a consistently high score (Figure 3A). In 2012, 63 genotypes from POP369 with multiple harvests exhibited different trends for FD scores, comprising 23 increasing, seven decreasing, 19 stable high and 14 stable low FD genotypes, as the season advanced (Figure 3B). POP356 in 2011 had 48 genotypes with multiple harvest, also exhibited four different trends where seven genotypes exhibited increasing, 12 genotypes decreasing and 29 genotypes did not show any variation of FD score between the harvest dates, with 24 genotypes scoring consistently low, and five exhibiting a consistently high score (Figure 3C). Over all in both populations across the years, over 50% genotypes were consistent in their trend of susceptibility either consistently low or consistently high.

In the POP369 population, weak correlations of r = 0.357 (P < 0.0001) and r = 0.27 (P < 0.0001) were observed between FD and harvest date in 2011 and 2012, respectively, although no such significant correlation was found in the POP356 population in 2011. Analysis of variance in the POP369 population indicated a significant effect of year, explaining 4% of the phenotypic variation in FD (P < 0.001), whilst the effect of genotype and harvest date accounted for a higher proportion of the phenotypic variation, at 54% (P < 0.0001) and 23% (P < 0.0001), respectively. Although interaction between genetics and harvest date was not significant, the effect of the genetics x year interaction accounted for 22% of the phenotypic variation in FD (P < 0.05).

### Fruit firmness and TSS

In 2011, TSS for both populations ranged from 10 to 13.5 °Brix. In 2012, TSS ranged from 9 to 14 °Brix for POP369. However, no significant correlation was observed between FD and TSS (Table 2). Fruit firmness ranged from 15 to 45 N for both populations in 2011, while in 2012 the fruit from the POP369 population had a slightly narrower range of 15 to 36 N. In 2012, fruit firmness showed a weak but significant (P < 0.01) correlation with FD for POP3569, however neither population POP369 or POP356 showed any significant correlation between FD and fruit firmness in 2011 (Table 2).

**Table 2:** **Correlation table (** ***r*** **) for all trait data in relation to harvest date, friction discolouration, total soluble solids and firmness**

		**POP369** **(2011)**			**POP369** **(2012)**				**POP356** **(2011)**			
**Trait**	**Harvest date**	**FD**	**TSS**	**Firmness**	**Harvest date**	**FD**	**TSS**	**Firmness**	**Harvest date**	**FD**	**TSS**	**Firmness**
**Friction discolouration**	0.36**				0.27**				ns			
**Total soluble solids**	ns	ns			−0.34**	0.16*			ns	ns		
**Firmness**	ns	ns	0.29**		−0.14*	−0.21**	0.16*		ns	ns	ns	
**Polyphenol oxidase**	−0.36**	ns	ns	ns	0.15*	0.20**	0.28**	ns	−0.25**	ns	ns	ns
**Ascorbic acid**	0.31**	ns	ns	ns	ns	ns	ns	−0.15*	0.54**	ns	ns	ns
**Chlorogenic acid**	−0.19*	−0.27**	0.18*	ns	−0.31**	ns	0.2**	ns	−0.18**	−0.12*	ns	0.27**
**Cryptochlorogenic acid**	ns	−0.27**	ns	ns	−0.25**	ns	ns	ns	−0.2 **	ns	ns	0.21**
**Neochlorogenic acid**	ns	ns	ns	ns	−0.20**	ns	ns	ns	ns	ns	ns	0.22**
**Catechin**	ns	ns	0.21*	0.44**	−0.19**	−0.21**	0.15*	0.30**	ns	−0.20**	ns	0.27**
**Epicatechin**	ns	ns	ns	0.42**	ns	−0.15*	0.30**	0.40**	ns	−0.18**	ns	0.15*
**Procyanidin B2**	ns	−0.23**	0.19*	0.27**	−0.18*	ns	0.19**	0.22**	ns	−0.24**	ns	0.21**
**Isorhamnetin 3**-**galactoside**	−0.22*	ns	ns	ns	−0.21**	−0.15*	ns	ns	ns	ns	ns	0.19**
**Isorhamnetin rutinoside**	−0.37**	−0.25**	ns	ns	−0.30 **	ns	ns	ns	ns	ns	ns	0.23**
**p**-**coumaryl quinic acid**	−0.21*	−0.2*	0.27**	0.23**	−0.23**	ns	0.20**	ns	ns	ns	ns	ns
**Quercetin galactoside**	−0.17*	ns	ns	ns	−0.18**	−0.15*	ns	0.23**	−0.13*	ns	ns	ns
**Quercetin glucoside**	−0.25**	ns	ns	ns	−0.32**	−0.20**	0.19**	0.25**	−0.20**	ns	ns	ns
**Quercetin arabinoside**	−0.22**	−0.21*	ns	ns	−0.24**	ns	ns	ns	ns	ns	ns	0.16**
**Quercetin rhamnoside**	−0.25**	−0.19*	ns	ns	ns	ns	ns	ns	ns	ns	ns	ns
**Quercetin rutinoside**	−0.28**	ns	ns	ns	−0.27**	−0.19**	ns	0.16*	−0.20**	ns	ns	ns
**Quercetin**	0.34**	0.22**	ns	ns	ns	−0.17*	ns	ns	0.16**	ns	ns	0.37**
**comp**_**417.12** **(1)**	−0.20*	−0.21*	ns	ns	−0.24**	ns	ns	ns	ns	ns	ns	0.17**
**comp**_**417.12 ** **(2)**	ns	−0.22**	ns	ns	−0.24**	ns	ns	ns	ns	ns	ns	0.15*

Data from POP369 were collected in two successive years (2011 and 2012) while POP356 was analysed in 2011 only. N.B.: comp_417.12 (1) and comp_417.12 (2) are unknown compounds identified from LC-MS quantification analysis, represented by their molecular weight. Units for trait studied are following: FD (scale), TSS (°Brix), Firmness (N: newton), PPO (change in A_420_/ g/minute), AsA and polyphenols compounds (concentration). comp_417.12 (1) and comp_417.12 (2) are unknown polyphenol compounds identified from LC-MS quantification analysis, represented by their molecular weight.

Note: * = P < 0.05 ** = P < 0.01 and ^ns^ = non-significant.

### Polyphenolic compound, AsA concentration and PPO activity in pear segregating populations

A subset of 17 polyphenol compounds was identified by using the QuantAnalysis software. This subset included flavanols, flavonols, procyanidins, two unknown compounds [417.12 (1) and 417.12 (2)] and chlorogenic acid. Chlorogenic acid was the most abundant polyphenol found in pear fruit peel in both populations in both years. The concentration of all 17 polyphenolic compounds varied among individual progeny in both populations, and showed significant correlation with FD for some compounds, however none showed high correlation values with FD (Table 2). Overall these compounds are negatively correlated with FD, as is clear in Table 2.

Although individual progeny exhibited a wide range of PPO activity in fruit for both years, PPO activity was weakly correlated with FD for POP369 in 2012 only. A weak yet significant negative correlation between concentration of some of the polyphenol compounds and PPO activity was observed in the 2012 data from population POP369, where PPO activity and epicatechin concentration exhibited a significant (P < 0.01) correlation (r = −0.28). AsA concentration showed a significant correlation with harvest date for both populations in 2011 and no correlation in 2012 for POP369 (Table 2). No significant correlation was observed between FD and AsA concentration.

### Genetic map construction

Parental genetic maps were constructed for POP369 and POP356 populations using a subset of 1144 and 1357 polymorphic SNPs, respectively. The genetic maps for QTL analysis were modified from the maps described in [47], by removing dominant markers with the segregation ratio 3:1 in order to improve their utility for QTL mapping. Numbers and segregation types of polymorphic, mapped, and revised for QTL map markers are provided in Table 3 and for detailed maps used for QTL analysis see Additional file 2: Figure S2. The revised parental maps of the POP369 population comprised 173 and 265 markers for the male and female parents, respectively. The POP369-male parental map spanned 858.2 cM (one SNP every 4.9 cM) over 23 groups across the 17 LGs, of which LGs 2, 9, 11, 12, 13, 14 and 17 were split into two parts. The POP-369 female parental map spanned 1027.9 cM (one SNP every 3.3 cM) over 20 groups across the 17 LGs, of which LGs 10 and 13 were split into two, and LG5 into three parts. The map of POP356-female consisted of 398 markers covering 885.9 cM and had 28 groups across the17 LGs, with 202 markers in common with the POP369-female map. The POP356-male map comprised 353 SNPs covering 1114.6 cM and spanned 23 groups across the 17LGs (Additional file 2: Figure S2a, b).

**Table 3:** **Number and segregation type of markers in QTL maps of the POP369 and POP356 pear populations**

		**POP369**						**POP356**			
**Marker segregation**	**Pear SNPs**	**Apple SNPs**	**Total**	**Marker segregation**	**Pear SNPs**	**Apple SNPs**	**Total**	**Marker segregation**	**Pear SNPs**	**Apple SNPs**	**Total**
ABxAA/BB	144	69	213	00xA0/00xB0/BBxB0	18	96	114	ABxAA/BB	90	95	185
ABxAB	16	37	53	A0xA0/B0xB0	3	31	34	ABxAB	92	51	143
BB/AAxAB	8	37	45	A0x B0	1	2	3	BB/AAxAB	97	127	224
				A0x AB/B0xAB/ABxB0	3	2	5				
Total	168	143	311	Total	25	131	156	Total	279	273	552

### Scope of QTLs identified for genetic control of fruit traits

QTLs were detected for 22 fruit traits, including FD score (Table 4), TSS, fruit firmness, PPO activity, AsA concentration and LC-MS peak area (response/min) for 17 polyphenolic compounds. A total of 105 QTLs with significance of P < 0.005 were detected for the 22 traits over two years for the POP369 population (Additional file 3: Table S1), and 77 QTLs for the POP356 population (Additional file 4: Table S2). The largest cluster, which comprised 22 QTLs associated with fruit firmness, PPO activity, concentration of AsA and five polyphenolic compounds (catechin, epicatechin, procyanidin B2, isorhamnetin rutinoside and quercetin), was identified on LG3 in POP369 for both parents. The largest cluster for both parents of population POP356 is located on LG5, with 11 QTLs associated with the concentration of polyphenolic compounds [isorhamnetin galactoside/glucoside, quercetin arabinose/rhamnoside and compounds 417.12(1) and 417.12(2)].

**Table 4:** **Quantitative Trait Loci** (**QTL**) **detected for friction discolouration (FD) in POP369 and POP356 pear populations**

**Year**	**Data type**	**Parent**	**LG**	**Position**	**SNP marker**	**Kruskal**-**Wallis** (K*)	**Significance**	(%) **Variance**
2011	average	POP369- female	2	59.44	nullss475883075	8.9	P < 0.005	11.37%
2011	average	POP369- female	15	6	ss527788075	6.3	P < 0.05	10.26%
2011	average	POP369- female	14	4.93	ss527788030	6.1	P < 0.05	6.22%
2011	average	POP369- female	3	48.09	ss527788418	5.8	P < 0.05	8.30%
2011	max	POP369- female	2	59.44	nullss475883075	10.1	P < 0.005	12.09%
2011	max	POP369- female	16	42.79	nullss475878310	6.7	P < 0.01	7.30%
2011	max	POP369- female	14	4.93	ss527788030	5.1	P < 0.05	5.50%
2011	max	POP369- female	3	48.09	ss527788418	5.0	P < 0.05	6.86%
2012	average	POP369- female	3	26.02	ss527788282	6.2	P < 0.05	8.74%
2012	average	POP369- female	14	8.8	ss527788968	3.7	P < 0.1	8.16%
2012	max	POP369- female	3	26.02	ss527788282	9.0	P < 0.005	12.85%
2012	max	POP369- female	14	4.93	ss527788030	3.8	P < 0.1	5.72%
2012	max	POP369- female	10	36	nullss475879653	3.5	P < 0.1	3.48%
2011	average	POP369- male	2	12.09	nullss475877109	8.2	P < 0.005	10.05%
2011	average	POP369- male	14	3.4	ss527789200	6.9	P < 0.01	8.92%
2011	average	POP369- male	13	2.75	ss475882576	5.1	P < 0.05	6.07%
2011	max	POP369- male	2	12.09	nullss475877109	9.1	P < 0.005	10.70%
2011	max	POP369- male	14	3.4	ss527789200	7.1	P < 0.01	9.15%
2011	max	POP369- male	16	16.29	nullss475878313	5.7	P < 0.05	6.29%
2011	max	POP369- male	13	2.75	ss475882576	5.3	P < 0.05	6.74%
2011	max	POP369- male	7	16.79	ss475878863	5.2	P < 0.1	9.07%
2012	average	POP369- male	7	42.04	nullss475876200	8.0	P < 0.005	8.34%
2012	average	POP369- male	4	25.6	ss475876768	7.3	P < 0.01	8.68%
2012	max	POP369- male	7	42.04	nullss475876200	7.0	P < 0.01	8.67%
2012	max	POP369- male	2	20.08	ss475877562	6.7	P < 0.05	8.34%
2012	max	POP369- male	4	25.59	ss475876768	6.7	P < 0.01	7.16%
2012	max	POP369- male	9	9	ss527787770	4.3	P < 0.05	5.02%
2011	average	POP356- female	11	23.60	ss527788944	12.6	P < 0.005	9.70%
2011	average	POP356- female	15	3.44	ss527789584	8.34	P < 0.05	8.89%
2011	average	POP356- female	5	0	ss475879840	6.8	P < 0.01	4.37%
2011	max	POP356- female	11	2.96	ss475880309	13.3	P < 0.005	10.46%
2011	max	POP356- female	15	3.44	ss527789584	8.62	P < 0.05	8.33%
2011	average	POP356- male	11	20.60	ss527788944	12.6	P < 0.005	9.70%
2011	average	POP356- male	2	3.17	ss527788737	8.52	P < 0.005	6.00%
2011	average	POP356- male	15	85.81	ss527789303	8.3	P < 0.05	8.89%
2011	average	POP356- male	16	104.88	ss527789436	7.4	P < 0.01	6.94%
2011	max	POP356- male	11	20.60	ss527788944	13.4	P < 0.005	10.17%
2011	max	POP356- male	2	3.17	ss527788737	8.7	P < 0.005	7.52%
2011	max	POP356- male	15	85.81	ss527789303	8.6	P < 0.05	8.33%

QTLs were identified using average and maximum FD score from multiple harvests of the same seedling. SNPs are presented using the NCBI dbSNP accession number (ss#). Apple SNPs are represented with an accession number starting with ‘4’ while pear SNPs accessions start with ‘5’.

### QTL for friction discolouration of fruit

**Figure 4:**
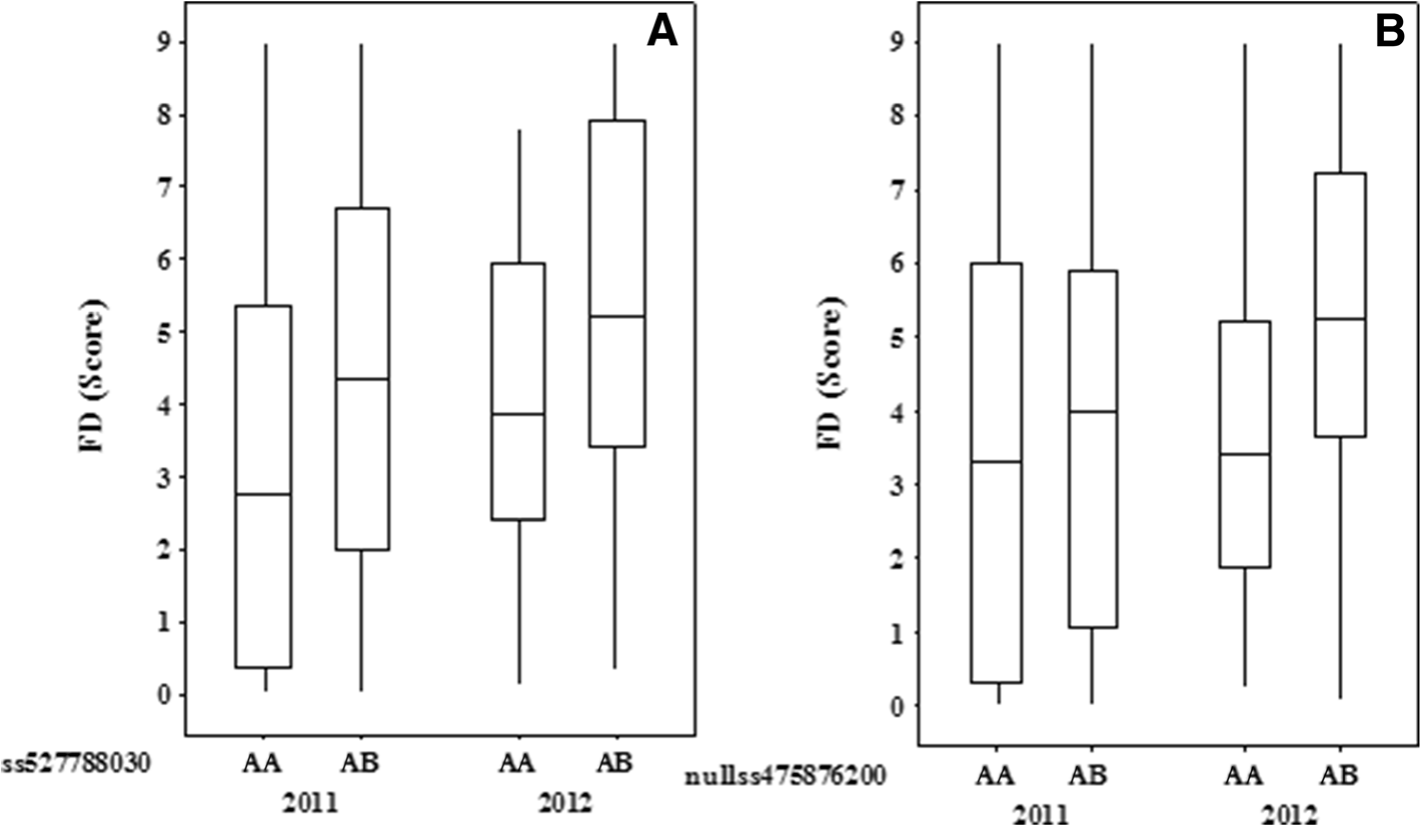
**Graphical representation of stable QTL controlling fiction discolouration (FD) across the years. A)** represents stable QTL for POP369-female parent on LG14 and **B)** represents QTL on LG7 from POP369-male parent.

**Figure 5:**
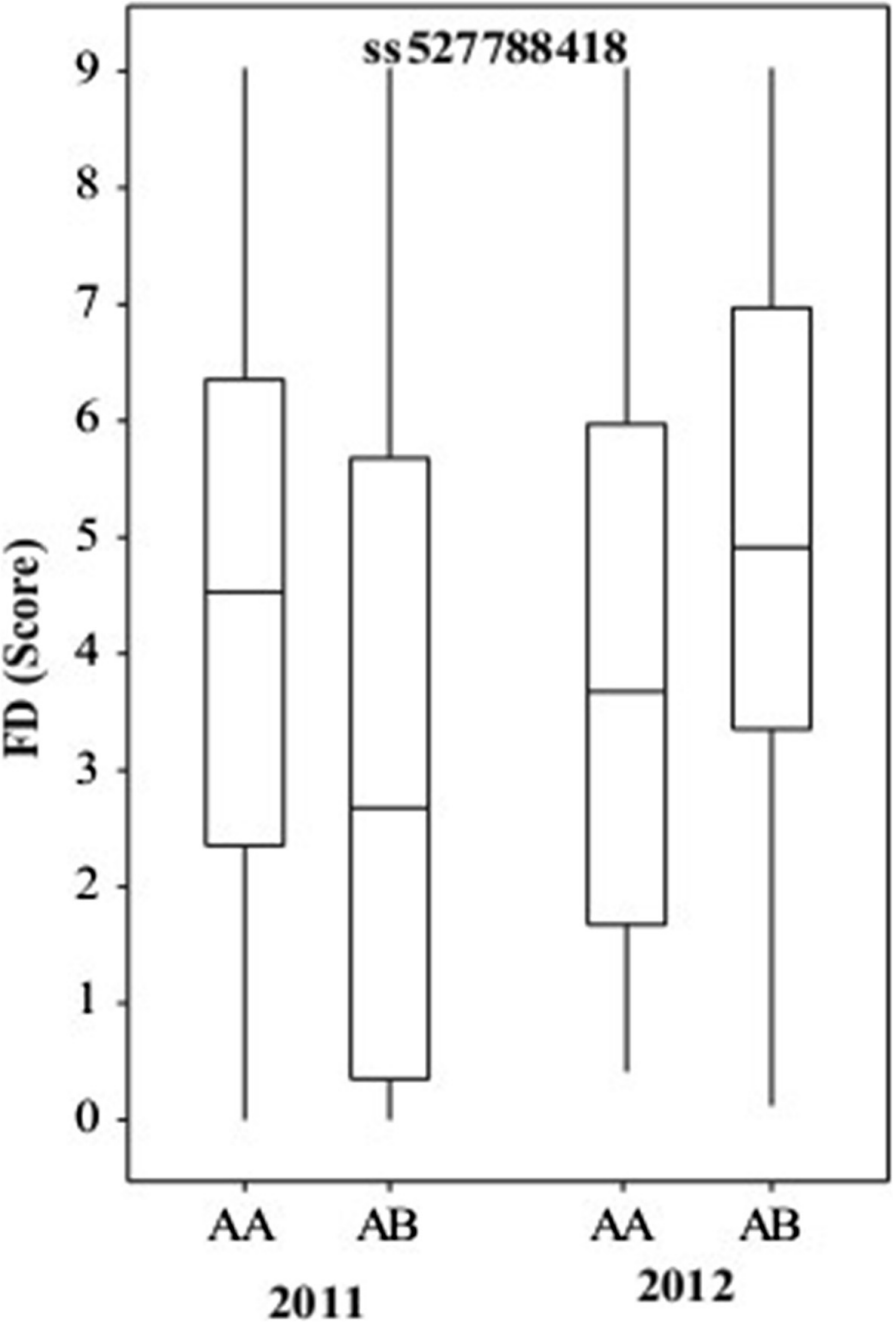
**Friction discolouration (FD) QTL on LG3 for parent POP369**-**female**; **opposing allelic trend in 2011 and 2012.**

As FD was non-normally distributed in both populations (Additional file 5: Figure S3), the Kruskal-Wallis test was used for QTL analysis. A total of 27 QTLs over 10 chromosomal regions (LGs 2, 3, 4, 7, 9, 10, 13, 14, 15 and 16) were detected for FD, using the average and maximum score of multiple harvests in 2011 and 2012 for population POP369 (Table 4), with the proportion of genotype explained by each QTL ranging from 3.5% to 13%. In general, the QTLs in common were for average and maximum FD scores (Table 4). The QTL detected on LG14 derived from the POP369-female parent was stable between years when either the maximum or average FD score data classes were used, with the homozygous AA genotype for marker ss527788030 linked to low FD score (Figure 4A). The QTL on LG7 of the POP369-male parent was not stable between years, as it only exhibited a strong effect in 2012, however a weaker effect QTL in 2011 was identified in another location of the same LG for the same parent. The homozygous AA genotype for marker nullss475876200 from LG7 was linked to low FD score in 2012 (Figure 4B). The marker information from QTLs on LG7 and LG14 from POP369 was combined into four possible genotypic combinations (Table 5) and compared with phenotype data from those multi-harvest date seedlings categorised into the four FD groups shown in Figure 3A,B (i.e. consistently high and low FD score, increasing and decreasing FD score with advancing harvest). In 2012, seedlings lacking both LG7 and LG14 QTLs (AB genotype for both SNP markers) exhibited a consistently high FD score (10), increasing (22) or decreasing (4) with late harvest date, with none that showed a consistently low FD. However, the seedlings with genotypes associated with low FD for both QTLs (AA genotype for both markers) had consistently low (6), decreasing (6) and increasing (9) FD scores during the season, while there were no seedlings with consistently high FD. The trend was not as clear in 2011, probably due to the weaker effect of the LG7 QTL in this year however, four seedlings having consistently high FD scores also lacked the low FD QTL genotypes for both LG7 and LG14. Another FD QTL for POP369-female was located on LG3, however, the allelic trend was inconsistent between years (Figure 5).

**Table 5:** **Genotypic effect of the friction discolouration (FD) QTLs detected in the POP369 population in 2011 and 2012**

		**2012**				**2011**			
**LG14**	**LG7**	**Consistent high**	**Consistent low**	**Increasing**	**Decreasing**	**Consistent high**	**Consistent low**	**Increasing**	**Decreasing**
AA (+)	AA (+)	0	6	9	6	0	2	2	0
AA (+)	AB	23	16	16	0	0	11	4	2
AB	AA (+)	6	10	9	2	0	8	0	0
AB	AB	10	0	22	4	4	8	2	2

Seedlings are grouped according to their seasonal trend for FD susceptibility as illustrated in Figure 3. The markers with the most significant Kruskal-Wallis value were used (Table 4): ss527788030 and nullss475876200 for LG14 and LG7, respectively. Alleles favourable for a low FD score are marked with a “+”.

In total, 12 QTLs over five chromosomal regions (LGs 2, 5, 11, 15 and 16) were detected in the POP356 population using the average and maximum FD score, with genotypic variation explained ranging from 4.4% to 10.5% (Table 4). The QTLs on LGs 11 and 15 were common to both parental maps in the POP356 population. Common QTLs between populations are located on LG2 of the POP356-male parent and both parents of population POP369, however, for POP369-female parent this QTL was observed in 2011 only (Table 4).

### QTLs for fruit firmness, TSS, PPO activity and AsA concentration

A QTL linked to fruit firmness identified at the top of LG3 for both parents of both populations was stable between 2011 and 2012 for the POP369-male parent. Although TSS exhibited no stable QTL between years, TSS QTLs on LGs 2 and 16 were detected for both parents of POP369 in 2012. A QTL associated with PPO activity identified on LG3 of POP369-male was stable across the years and was detected only in 2012 in the POP369-female parent. The POP356 population had a QTL for PPO activity on LG2 for both parents, however, no QTL was detected on LG3 as for POP369 (Additional file 3: Table S1). Other QTLs associated with PPO activity that were unstable between years were located on LGs 5, 9 and 14 for POP369, and LGs 6 and 17 for POP356. QTLs influencing fruit AsA concentration were identified on LG3 of all four parental maps in 2011 only (Additional file 3: Table S1 and Additional file 4: Table S2).

### QTLs for polyphenolic compound concentration

A total of 86 and 64 QTLs were detected that were associated with the concentration of 17 polyphenolic compounds in pear fruit for POP369 and POP356, respectively. QTLs detected for polyphenols were identified on all LGs, except LG 4, 6 and 10 for population POP369, and LG 4, 13 and 16 for population POP356 (Additional file 3: Table S1 and Additional file 4: Table S2). The largest clusters of QTLs associated with polyphenol concentration were located on LG3 of POP369 and LG5 of POP356.

### QTL stability between years and parents

Additional file 3: Table S1 and Additional file 6: Figure S4 show that major stable QTLs exhibited across the years for the POP369-male parent were for control of fruit firmness, and PPO activity on LG3, as well as concentration of chlorogenic acid on LG9, catechin on LG3 and LG9, epicatechin on LG3, quercetin arabinose and unknown compounds 417.12(1) and 417.12(2) on LG5. QTLs that were stable across 2011 and 2012 in the POP369-female parent were associated with concentration of chlorogenic acid and cryptochlorogenic acid on LG1, catechin on LG17, epicatechin on LG3 and LG14, and procyanidin B2 on LG14. Although QTLs for chlorogenic acid and cryptochlorogenic acid were identified in both years at the same location, in 2011 the K value (P < 0.01) was lower than the set threshhold (P < 0.005).

Clusters of QTLs that were identified on LG3 and associated with fruit firmness and epicatechin concentration were stable between 2011 and 2012, and between parents of each of the two populations as well as across these populations. In addition, for population POP369, several other QTLs were conserved between parents, however, were identified in one year only. Examples for 2012 include: QTLs on LG2 and LG16 for control of TSS, chlorogenic acid concentration on LG9, catechin on LG3, and procyanidin B2 on LG3. QTLs associated with iso-rhamnetin galactoside/glucoside concentration were observed on LG2 in 2011 only, as well as quercetin on LG3 and LG15. Population POP356 also exhibited QTLs conserved between the parents: control of fruit firmness on LG3, PPO activity on LG2, concentration of AsA on LG3, concentration of cryptochlorogenic acid on LG9, catechin and epicatechin on LG3, procyanidin on LG15, iso-rhamnetin galactoside/glucoside on LG5 and LG6, iso-rhamnetin rutinoside, quercetin galactoside and quercetin arabinose on LG5, quercetin rhamnoside on LG3, quercetin rutinoside on LG2 and LG7, quercetin on LG12, and unknown compounds 417.12(1) and (2) on LG5.

### QTL co-location between traits

**Figure 6:**
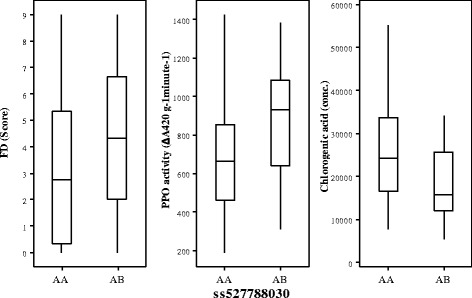
**Common QTLs controlling friction discolouration (FD) and other variables on LG14 in 2011.**

In total, 10 genomic regions exhibited QTLs for different fruit traits that co-located (Additional file 3: Table S1 and Additional file 4: Table S2; Additional file 6: Figure S4). A QTL located on LG14 of POP369-female was for FD, PPO activity and chlorogenic acid concentration in 2011 (Figure 6). For POP369-male parent stable QTL for epicatechin and procyanidin B2 is also located at the same location of LG14. For POP369-male, QTLs associated with firmness, PPO activity, and concentration of catechin and epicatechin in both years and procyanidin B2 in 2011 only, co-located on LG3 for both 2011 and 2012. For the POP356-female parent, QTLs co-locating at LG3 are associated with fruit firmness and concentration of AsA, catechin, epicatechin and quercetin rhamnoside. Similar group of QTLs was also detected for POP356-male parent on LG3 (Additional file 6: Figure S4). QTLs controlling concentration of the flavanols isomers (catechin and epicatechin) were identified on LG3 in the same genomic location across the populations and between the two years of the study, except for POP369-female, where a potential QTL identified for catechin in 2011 was lower than the set threshold (i.e. P < 0.01). This parent also exhibited stable QTLs on LG14 for epicatechin and procyanidin B2 between the two years. Parent POP369-male exhibited QTLs for catechin and epicatechin on LG3 across both years, and for procyanidin B2 only in 2012 on LG3, while the POP369-female parent exhibited QTLs on LG3 for epicatechin in both years, and in 2012 only for epicatechin and procyanidin B2. Potential QTLs associated with concentration of catechin and procyanidin on LG3 were detected in 2011, however, the significance was lower than the set threshold (P < 0.01) (data not shown). In POP356, both parents exhibited QTLs on LG3 associated with concentration of catechin and epicatechin, but not for procyanidin B2.

## Discussion

### The relationship of FD to fruit maturity

FD in pear is recognised as a complex postharvest disorder that is highly influenced by both genetic and environmental factors (growing area, season etc.), as well as those related to development (harvest maturity). Our study supports this view, based on clear variation in susceptibility to FD, not only among the seedlings of the two segregating populations but also among fruit from the same seedling. This indicates that, while the genetic component of FD control is significant, there is also a substantial non-genetic effect. The significant correlation of FD to harvest date in population POP369 emphasizes the role of fruit maturity in the development of this disorder. Harvest of fruit both before and after optimal maturity can increase the potential for development of FD. Kvåle [48] reported that fruit harvested before the climacteric peak are more susceptible to FD than are late harvested fruit. However, this hypothesis was later contradicted by Burger et al. and Mitcham et al. [50,53], who found that late harvested fruit were more susceptible to FD. We noted these opposing trends in the interspecific segregating populations in our own study, as results from POP369 backed up the observation that late harvested fruits are more prone to FD, whereas POP356 exhibited the opposite trend, indicating that the effect of fruit maturity is a variable that is based on genetics. Our study hence confirms the hypothesis by Burger et al. [50] that the relationship between FD and fruit maturity is not always consistent among genotypes. In their study involving two pear genotypes only, ‘Packham’s Triumph’ showed greater susceptibility to FD at late harvest compared with earlier harvest, while ‘Comice’ exhibited the opposite trend. Our analysis using the variation among hundreds of individual genotypes has highlighted even more clearly the complexity of the relationship between FD and fruit maturity.

Pear maturity indices are complex and generally not reliable across the species as European and Asian pear cultivars have different indices [59], which are not reliable for interspecific progeny between Asian and European type pear. The issue of optimum time of harvest is especially challenging for breeding populations obtained from interspecific Asian x European hybridisation. Although the indices currently employed to determine maturity for harvest (firmness, total soluble solid contents and ground colour) are the same as for apple, these indices may well differ between genotypes, orchards and/or seasons. In this study, fruit firmness and TSS were measured in an attempt to evaluate fruit maturity of selected genotypes. While these traits segregated in both progenies, no relationship with susceptibility to FD was identified. This finding underlines the need for development of accurate fruit maturity descriptors in pear, especially for individual new cultivars of interspecific hybrids.

### The potential relationship between FD and fruit polyphenol content

Polyphenols are specific substrates for PPO and participate in the browning mechanism underlying FD. Chlorogenic acid has been found to be the most abundant polyphenol present in pears [60–62] and we confirmed this finding in both segregating populations. Neither this nor any other of the fruit polyphenolic compounds quantified over two years in two segregating populations totaling 250 seedlings showed strong phenotypic correlation to FD (Table 2). This finding conflicts with previous reports, which demonstrated in a small number of commercial European pear cultivars that certain phenolic compounds, such as chlorogenic acid, act as a rate-limiting factor in FD incidence [4,48]. However, our experimental conditions utilising over 250 individual genotypes of a mixed *P. bretschneideri*, *P. pyrifolia* and *P. communis* heritage are very different to these earlier studies utilising a few commercial cultivars with known maturity and a much narrower *P. communis* species background. Pear has very distinct cultivar variation for susceptibility to FD, as some varieties are recognized to be more susceptible to FD than others that have lower or almost no tendency to develop FD [13]. Our statistical analysis of a large number of traits in over 250 individual genotypes from interspecific crosses strengthens the hypothesis that the relationship between FD and rate-limiting polyphenols cannot be generalised and therefore cannot be used as a selection criterion for new cultivar breeding. However, although the relationship between polyphenol compounds and FD may not hold true at the global phenotype concentration, it may be valid at the concentration of a QTL explaining part of the variation in a specific genetic background, justifying our decision to dissect and compare the genetic control of FD and other fruit traits by QTL analysis. Indeed, we found that FD QTLs co-located with QTLs governing other traits that had been suggested previously to be associated with susceptibility to FD.

### QTL co-location

Co-location of QTLs associated with different traits may mean that the QTLs for both traits are tightly linked, or even that the same gene controls both. In the second situation this helps provide a clue to as to the nature of the molecular control underlying both traits.

An example is the QTL located on LG14 of POP369-female parent that was common for FD, PPO activity and chlorogenic acid concentration (Figure 6; Additional file 3: Table S1) along with epicatechin and procyanidin B2. However QTL for PPO and chlorogenic acid were detected in 2011 only but this could explain the relationship between FD, enzyme (PPO) and substrate (chlorogenic acid). Although we did not identify any strong statistical correlation among the phenotypes considered as a whole (Table 2), when considering only the QTL cluster on LG14 of POP369-female, we observed that individuals in POP369 carrying the low FD allele exhibited both low PPO activity and a high concentration of chlorogenic acid (Figure 6). An opposing trend between chlorogenic acid and FD indicates that although the substrate amount is not crucial in terms of browning in interspecific pears, the PPO activity may play an important role. In this case, we could hypothesise that a candidate gene influencing PPO activity located in this genomic region of POP369-female parent, but not in POP369-male or POP356-male, might contribute to FD susceptibility via a stimulation of enzymatic browning in pear.

A second example is the QTL cluster for firmness, PPO, catechin and epicatechin detected between 0 and13 cM on LG3 of parent POP369-male (Additional file 3: Table S1; Figure S4). Polyphenols, such as catechin and epicatechin, are substrates for PPO during the enzymatic browning that characterises FD. Clearly, there are opportunities for further analysis, including mining the European [63] and Chinese [45] pear genome sequences in the QTL region to identify candidate genes. Although no candidate gene for the control of such compounds has been identified in the syntenic region on LG3 in the apple genome [22] to date, the apple genome is another clear source of information.

Polyphenol content of fruits and vegetables is dependent on fruit maturity, pre- and post-harvest operations as well as genetic characteristics [64,65], and firmness is one of the most reliable indicators of maturity in commercial European pear cultivars [66]. Our identification of a stable QTL on LG3 across years and populations, and in common for control of fruit firmness, PPO activity and polyphenol concentration, confirms reports of the physiological relationship between firmness and maturity in the accumulation of polyphenols [5,49,53].

Procyanidin B2 is an oligomeric compound, formed from catechin and epicatechin molecules and hence might be predicted to exhibit QTLs in the same region as catechin and epicatechin. Parent POP369-female exhibits similarly located QTLs for epicatechin and procyanidin B2 on LG14.

### Significance of stability of detected QTLs

Despite the complexity of the FD disorder and strong influence of environmental and developmental factors, we were able to identify 27 and 12 QTLs for POP369 and POP356, respectively (Table 4) by using average and maximum FD score. None of these QTLs can be regarded as a major QTL, as the strongest among them explains only 12.48% of the phenotypic variation. As we collected phenotypic data for the POP369 population in two consecutive seasons (2011 and 2012), QTLs could be verified for their stability across years. A stable QTL across the years 2011 and 2012 was identified on LG14 for parent POP369-female (Figure 4). Although the suggested FD QTL on LG7 was below the threshold of detection (Figure 4B) in 2011, a likely reason for lower significance could be the large environmental and developmental effect on FD incidence. Although a FD QTL is located on LG3 for POP369-female parent, the allelic trend is different between years (Figure 5), which implies that this QTL is an artefact. Population POP356 had only one year (2011) of phenotypic data, so it is not possible to verify QTL stability across years. However, this rather inbred population exhibits genomic regions on LG11 and LG15 that are conserved between its parents (Table 3).

Our QTL analysis indicated that FD is a polygenic trait controlled by many small effect QTLs, of which only a subset are stable across years. The QTLs on LG7 and LG14 provide closely linked markers, which are candidates that might be theoretically used for MAS. However, these QTLs individually explain only 8% of the phenotypic variation, which would provide only limited genetic gain if they were used for selection in a breeding population. Also, when the QTLs are considered in combination, none of the seedlings with the marker genotype associated with low FD exhibited a consistently high score for FD in 2012 and 2011. No seedlings with the other homozygous AB type allelic pair appeared to have a consistently low FD score in 2012, but in 2011, eight seedlings of this group exhibited a consistently low score (Table 5). These results point towards the possibility of using these QTLs in combination for MAS in bi-parental populations.

The polygenic control of FD by small effect QTLs suggests that genomic selection may be a more suitable approach to cull susceptible seedlings early in the breeding cycle. Genomic selection makes use of genome wide markers to predict total genetic value instead of phenotype and has been evaluated recently in apple [67]. In genomic selection, a prediction equation is established from genotype and phenotype data collected from the ‘training population’ and this prediction equation is used later to estimate genomic estimated breeding values (GEBVs) of individual progeny in the ‘selection population’ [68].

A number of QTLs for other fruit traits were stable between years. Parent POP369-male exhibited stable QTLs across years associated with fruit firmness, PPO activity and concentration of chlorogenic acid, cryptochlorogenic acid, catechin, epicatechin, quercetin arabinose, as well as the unknown compounds 417.12(1) and 417.12(2) (Additional file 3: Table S1). Some QTLs were stable across the years for population POP369 and were detected in both parents. For example, a stable QTL controlling fruit firmness was located on LG3 and was stable across years and detected in the same location in both parents of POP369 and POP356. Likewise, another QTL associated with chlorogenic acid concentration was identified on LG9 for parents of both populations and was stable across both years (Additional file 3: Table S1 and Additional file 4: Table S2).

From epidemiological studies, there is evidence that consumption of dietary anti-oxidants through eating polyphenol-rich fruits and vegetables can enhance cellular defence and help to guard against diseases, such as cancer, coronary heart disease and osteoporosis. Chlorogenic acid has strong anti-oxidant properties and is the most abundant type of polyphenol in pear. Breeders could use this QTL associated to chlorogenic acid to select genotypes rich in this compound. Furthermore, candidate genes controlling fruit firmness in pear might be identified by utilising the stable QTL on LG3, to identify candidate genes in the aligned genome sequences of both Chinese and European pear, as well apple.

### QTLs orthologous between apple and pear

Pear belongs to the Pyreae subfamily of the Rosaceae, which also includes apple, and their genomes are syntenic [69]. Syntenic species conserve QTLs for similar traits and this synteny information opens new possibilities for identification of candidate genes controlling similar traits across species. QTLs associated with concentration of chlorogenic acid and its isoforms, i.e. cryptochlorogenic acid and neochlorogenic acid, located on LG17 of the POP356-female parent are orthologous to a QTL identified for control of chlorogenic acid concentration in apple [22]. Interestingly, in the POP369 population both parents have QTLs for the same variables on LG9, which is homeologous to LG17 in apple [70] and pear [45]. This homology in the *Malus* and *Pyrus* genomes indicates that these QTLs may be derived from paralogous gene copies from the Pyreae whole genome duplication [70]. In apple, a QTL for chlorogenic acid is also located at the bottom of LG17, where the *HCT*/*HQT* (hydroxy cinnamate transferase/hydroxy quinate transferase) gene is located [22]. The *Pyrus HCT*/*HQT* gene is therefore a strong candidate gene for the LG17 QTL from POP369-female.

A stable QTL governing pear fruit firmness is located on LG3 in the same region where a QTL for apple firmness has been detected previously [71], however, no apple candidate gene has yet been proposed for this QTL.

## Conclusions

Unlike other more studied fruit species, such as tomato and apple, genetic information about the control of expression of pear fruit characters has been scanty to date. Of four reported QTL mapping studies in pear (two European, one Chinese and one interspecific between European and Chinese pear), only one concerns fruit traits [34]. None of these studies employed gene-rich and SNP-based genetic maps. These new generation maps provide advantages as they are derived from the *Pyrus* genome sequence and hence identified QTLs can be used to detect the candidate genes for these traits in the genome sequence. We have utilised the first SNP-based genetic maps in pear [47] to identify QTLs for 22 variables, including FD, using two interspecific segregating populations (POP369 and POP356). QTL clusters were found for all 22 variables with a number of QTLs being stable across years, parents and populations. Our QTLs associated with fruit firmness and concentration of AsA and polyphenolic metabolites are the first reported for pear. Most notably, the QTLs we detected that influence susceptibility to FD are the first fruit disorder QTLs to be reported in a tree species. This study clearly demonstrates that this postharvest disorder is controlled by multiple small effect QTLs, unlike fruit quality attributes, such as firmness and skin biochemical composition that are controlled by small and medium effect QTLs. In future, candidate genes for QTLs controlling firmness, PPO activity, and polyphenolic compound concentration will be identified utilising the reference genome sequences of pears ‘Bartlett’, ‘Dangshansuli’ and syntenic apple ‘Golden Delicious’. This will assist fruit biologists, postharvest scientists and pear breeders to develop an understanding of the genetic control of this highly challenging postharvest disorder. The polygenic nature of FD genetic control indicates that it will be difficult to apply marker-assisted selection, however, genomic selection could be employed to select elite genotypes with lower or no susceptibility to FD early in the breeding cycle. Our results also point towards the need for better fruit maturity estimation to avoid the noise in phenotypic data.

## Additional files

Additional file 1: Figure S1. Alignment of male parent from POP369 and POP356 to reference map with simple sequence repeat markers. Linkage group with notation ‘n’ represents reference map, SSR markers are represented by red colour. Additional file 2: Figure S2. Genetic linkage maps of POP369 and POP356 used for QTL analysis (Word file). **Figure S2a.** Genetic linkage maps of male and female parents of POP369. **Figure S2b.** Genetic linkage maps of male and female parents of POP356. Additional file 3: Table S1. List of QTLs controlling fruit traits except FD, for POP369. The Kruskal Wallis test was adopted to identify the QTLs, as almost all traits were non-normal in distribution. QTLs for each trait are given for each parent and for both years. SNPs are presented using the NCBI dbSNP accession number (ss #). Apple SNPs are represented with an accession number starting ‘4’ while pear SNPs accessions start with ‘5’. Additional file 4: Table S2. List of QTLs controlling fruit traits except FD, for POP356. The Kruskal Wallis test was adopted to identify the QTLs as almost all traits were non-normal in distribution. QTLs for each trait are given for each parent and for both years. SNPs are presented using the NCBI dbSNP accession number (ss #). Apple SNPs are represented with an accession number starting ‘4’ while pear SNPs accessions start with ‘5’. Additional file 5: Figure S3a. Graphical representation of distribution of trait data for POP369 in 2011 (Word file). **Figure S3b.** Graphical representation of distribution of trait data of POP369 in 2012. **Figure S3c.** Graphical representation of distribution of trait data of POP356 in 2011. Additional file 6: Figure S4. Linkage groups with stable QTLs in POP369 and POP356.
