# Supplementary material for: Genetic, metabolite and developmental determinism of fruit friction discolouration in pear
Source: BMC Plant Biol. 2014 Sep 16;14:241. doi: 10.1186/s12870-014-0241-3 (PMC4177423; doi:10.1186/s12870-014-0241-3)
Supplement: Additional file 1: Figure S1. — Alignment of male parent from POP369 and POP356 to reference map with simple sequence repeat markers. Linkage group with notation ‘n’ represents reference map, SSR markers are represented by red colour. [file 12870_2014_241_MOESM1_ESM.docx]

**Figure S1: Alignment of male parent from POP369 and POP356 to reference map with simple sequence repeat (SSR) markers.** Linkage group with notation ‘n’ represents reference map, SSR markers are represented by red colour.
