# Supplementary material for: Genetic, metabolite and developmental determinism of fruit friction discolouration in pear
Source: BMC Plant Biol. 2014 Sep 16;14:241. doi: 10.1186/s12870-014-0241-3 (PMC4177423; doi:10.1186/s12870-014-0241-3)
Supplement: Additional file 2: Figure S2. — Genetic linkage maps of POP369 and POP356 used for QTL analysis (Word file). Figure S2a. Genetic linkage maps of male and female parents of POP369. Figure S2b. Genetic linkage maps of male and female parents of POP356. [file 12870_2014_241_MOESM2_ESM.docx]

**Figure S2a: Genetic linkage maps of male and female parents of POP369**

Figure S2b: Genetic linkage maps of male and female parents of POP356
