# Supplementary material for: Genetic, metabolite and developmental determinism of fruit friction discolouration in pear
Source: BMC Plant Biol. 2014 Sep 16;14:241. doi: 10.1186/s12870-014-0241-3 (PMC4177423; doi:10.1186/s12870-014-0241-3)
Supplement: Additional file 5: Figure S3a. — Graphical representation of distribution of trait data for POP369 in 2011 (Word file). Figure S3b. Graphical representation of distribution of trait data of POP369 in 2012. Figure S3c. Graphical representation of distribution of trait data of POP356 in 2011. [file 12870_2014_241_MOESM5_ESM.docx]

**Figure S3a: Graphical representation of distribution of studied traits data of POP369 in 2011****Figure S3b: Graphical representation of distribution of studied traits data of POP369 in 2012**

**Figure S3c: Graphical representation of distribution of studied traits data of POP356 in 2011**
