# Supplementary material for: Genetic, metabolite and developmental determinism of fruit friction discolouration in pear
Source: BMC Plant Biol. 2014 Sep 16;14:241. doi: 10.1186/s12870-014-0241-3 (PMC4177423; doi:10.1186/s12870-014-0241-3)
Supplement: Additional file 6: Figure S4. — Linkage groups with stable QTLs in POP369 and POP356. [file 12870_2014_241_MOESM6_ESM.docx]

**Additional file6; Figure S4: Linkage groups with stable QTLs in POP369 and POP356**
